# Inhibition of HDAC3 induces neuroprotection by activating the Npas4 signaling pathway following surgical brain injury in rats

**DOI:** 10.22038/ijbms.2025.80235.17372

**Published:** 2025

**Authors:** Haiping Gu, Yating Gong, Muyao Wu, Mengying Shi, Jiejie Yu, Hongfei Zhu, Ya-ming Sun, Baoqi Dang

**Affiliations:** 1 Department of Neurology, Zhangjiagang TCM Hospital Affiliated to Nanjing University of Chinese Medicine, Suzhou, China; 2 Department of Rehabilitation, Zhangjiagang TCM Hospital Affiliated to Nanjing University of Chinese Medicine, Suzhou, China; 3 Department of Anesthesiology, Zhangjiagang TCM Hospital Affiliated to Nanjing University of Chinese Medicine, Suzhou, China; 4 Department of Emergency, Zhangjiagang TCM Hospital Affiliated to Nanjing University of Chinese Medicine, Suzhou, China; 5 Department of Orthopaedics, The Affiliated Aoyang Hospital of Jiangsu University, Suzhou, China

**Keywords:** Autophagy, Brain injury, HDAC3, Inflammation, Npas4

## Abstract

**Objective(s):**

Histone deacetylase 3 (HDAC3) can acetylate histones, negatively regulating Neuronal Per-Arnt-Sim domain protein 4 (Npas4) and participating in various pathological processes of central nervous system lesions. However, the role of HDAC3 in early surgical brain injury (SBI) remains elusive. This study aimed to determine the role of HDAC3 in early rat SBI and its underlying mechanism.

**Materials and Methods:**

The SBI model was constructed using the right frontal lobotomy of adult male Sprague-Dawley rats. The effects of RGFP966, a specific HDAC3 inhibitor, were assessed by western blotting, immunofluorescence, neurological scoring, and fluoro-Jade C staining.

**Results:**

HDAC3 protein expression was up-regulated after SBI and peaked at 24 hr relative to the Sham group. RGFP966 application can significantly improve brain edema and neurological dysfunction 24 hr after SBI, enhance autophagy, and reduce inflammation. In addition, we observed that Npas4 expression increased in SBI rats and was further up-regulated after HDAC3 inhibition.

**Conclusion:**

HDAC3 plays a role in rats’ complex pathogenesis of SBI. HDAC3 inhibition imparts a protective role in early brain injury in SBI in rats by regulating autophagy and inflammation via up-regulation of Npas4.

## Introduction

In the course of neurosurgery, normal brain tissue in the adjacent area is inevitably damaged, which is known as surgical brain injury (SBI). Such injury can lead to a series of pathological processes, including brain edema, blood-brain barrier (BBB) destruction, inflammatory reaction, and nerve cell death in the surgical site (1, 2). Using animal models, SBI has been shown to increase brain inflammation and cerebral edema, thereby exacerbating brain damage (3). Studies have shown that autophagy can protect cells from excessive and persistent inflammation (4, 5); the inhibition of autophagy contributes to excessive neuroinflammation following brain injury (6). Inflammation plays an important role in SBI pathogenesis, and autophagy contributes to the protective effect of nerve injury (7).

Epigenetic modifications play a significant role in the pathophysiology of various diseases (8, 9). Histone acetyltransferases (HATs) and histone deacetylases (HDACs) are the main components of epigenetic mechanisms, which mutually antagonize each other to determine histone acetylation levels (10), thereby regulating gene expression without directly altering DNA sequences. HDACs are members of an 18-member superfamily that deacetylate specific lysine residues, causing chromatin nucleosomes to revert to transcriptional inhibition, thereby inhibiting gene expression (11, 12). Inhibition of HDACs results in the enhancement of histone acetylation, relaxation of chromatin, and gene expression (13). HDAC3, a member of the HDAC family, is the most highly expressed type I HDAC in the brain and is mainly located in the nucleus of neurons (14, 15). It has been reported that HDAC3 is up-regulated in various pathological states of the central nervous system, such as cerebral ischemia, craniocerebral trauma, and subarachnoid hemorrhage, and it has been proven to impart neurotoxic effects (16-18). Selective inhibition of HDAC3 may thus be an attractive approach in targeting SBI by increasing the resistance of the central nervous system to surgical injury. 

HDAC3 regulates the transcription kinetics of the Neuronal Per-Arnt-Sim domain protein 4 (Npas4) gene by changing the level of histone acetylation (19), which in turn negatively regulates Npas4 (20). Npas4 is mainly expressed in neuronal cells, is responsible for directly regulating a large number of activity-dependent genes, and is involved in promoting neuron survival (21, 22). Npas4 has been reported to be involved in the regulation of inflammation and autophagy of the central nervous system (23, 24), which is closely related to the pathogenesis of a wide range of diseases. However, the roles of Npas4 and HDAC3 in SBI remain unclear.

Hence, this study established an *in vivo* rat SBI model to study the expression of HDAC3 in the cerebral cortex. In addition, HDAC3-specific inhibitor RGFP966 was selected to initially explore whether inhibiting HDAC3 can reduce SBI damage through the Npas4 signaling pathway, affecting inflammation and autophagy. More importantly, we also explored the underlying mechanism of SBI by screening for novel targets for treating this complex disease.

## Materials and Methods

### Animals

All male Sprague-Dawley (SD) rats were purchased from the Zhaoyan (Suzhou) New Drug Research Center. We used 92 rats (8 weeks) in the experiments following a 7-day acclimatization period. Ninety of them were exclusively used in statistical analysis. All rats were fed at a 12-hr/12-hr light/dark cycle with controlled temperature and humidity. The rats were provided with plenty of food and water. All experimental protocols received approval from the Institute of Animal Care Committee of Zhangjiagang TCM Hospital, Affiliated with Nanjing University of Chinese Medicine (Zhangjiagang, China, protocol code 2020-68-1), and were conducted following guidelines of the National Institutes of Health Guide for the Care and Use of Laboratory Animals. The humane endpoints of the study included dyspnea, cyanosis, persistent convulsions, and severe hypothermia that could not be recovered by warming measures. 

### Study design and experimental groups

**Figure 1:**
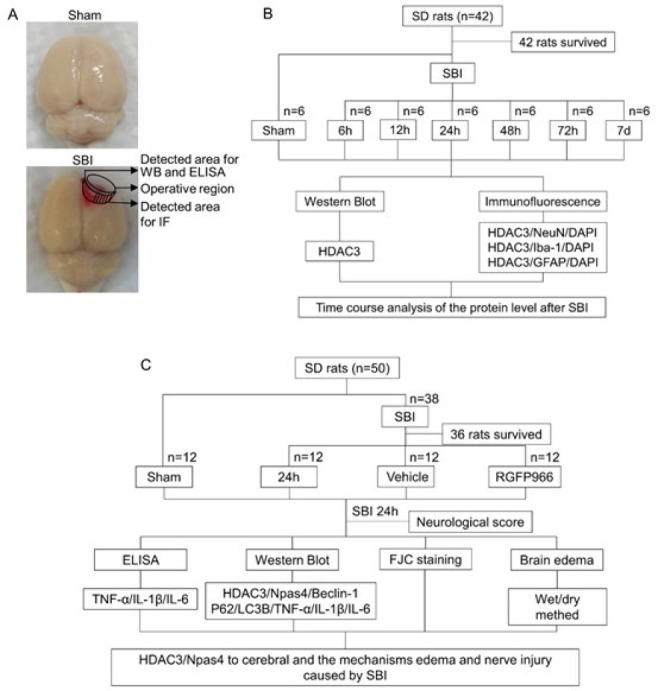
Experimental design

Experiment 1: No significant differences in body weight (280–320 g), week age, food intake, and exercise ability were observed. To determine the temporal expression profile of HDAC3 in Sprague-Dawley rats with SBI, 42 rats were randomly assigned into seven groups: a sham operation group and six groups of different time points after SBI, namely, SBI 6 hr, SBI 12 hr, SBI 24 hr, SBI 48 hr, SBI 72 hr, and SBI 7 days (Figure 1A). There were six rats in each group. The rats were sacrificed in each group at various time points and cortical samples <3 mm were collected at the edge of the damaged area. The right prefrontal cortex tissue in the same location as the SBI group was used for testing in the Sham group. These cortical tissues were used for Western blotting (WB) to assess HDAC3 expression and for double immunofluorescence analysis to evaluate localized HDAC3 expression (Figure 1B).

Experiment 2: To investigate the role of HDAC3 in SBI and its underlying mechanism, 48 rats (48 survivors out of 50, 2 sacrificed: 1 belonged to the SBI group and 1 belonged to the SBI + Vehicle group, did not recover from breathing arrest during modeling in SBI operation. This study had the same reasons for sacrifice and similar mortality rates as the previous studies (1, 25). They were randomly assigned to 4 groups: Sham, SBI, SBI+Vehicle, and SBI+RGFP966 (Figure 1C). There were 12 rats in each group. According to the results of experiment 1, rats in each experiment were sacrificed 24 hr after SBI, and brain tissues around the damaged area were collected. All groups were examined based on neurofunctional scores and then sacrificed. Twenty-four rats (six in each group) were used for ELISA, WB, and Fluoro-Jade C staining (FJC). The remaining 24 rats (6 rats in every group) were assessed in terms of brain edema (Figure 1C). The experiment was performed using the blind method; the analysts had no information on the type of samples being evaluated.

### Establishment of experimental rat SBI model

As previously reported, a rat model of SBI was used (25). SD rats were anesthetized via intraperitoneal injection of pentobarbital sodium (drug potency: 40 mg/kg). Upon anesthesia, the rats moved onto an operating table, and the skin on the top of the head was cleared and disinfected with aner iodine. Scalp, periosteum, and periosteum were incised and separated at the median sagittal line at the top of the skull, then 2 mm in front of the right anterior fontanelle and 2 mm beside the midline were used to make a bone window with a diameter of about 5 mm. The right exposed frontal lobe was resected, hemostasis was stopped, sterile saline was used to repeatedly flush the operative field until the flushing fluid was clear, disinfection was performed, and the wound was sutured. In the sham group, a similar surgical procedure was conducted without excision of the right frontal lobe following craniotomy, and then hemostatic suturing was performed (Figure 1B). Major parameters were monitored during and after the operation. According to the experimental design requirements, all rats were sacrificed at different time points.

### Drug injection

Intraperitoneal injection of RGFP966 (drug potency: 10 mg/kg in 10% DMSO; Biorbyt, US) was performed in SBI+RGFP966 group 30 min after SBI, once every 12 hr until sampling was obtained. Weight-matched SBI + Vehicles were injected with the same amount of 10% DMSO at the same time point. RGFP966 has been shown to be specific for HDAC3, effectively inhibiting HDAC3 activity, and is relatively effective in the distribution of RGFP966 in the central nervous system (26, 27).

### Tissue collection and sectioning

Experimental rats were induced to anesthesia by intraperitoneal injection of pentobarbital sodium at different time points after the operation. As previously reported (25), approximately 100 ml of 0.9% normal saline at 4 ^°^C was injected into the heart of the experimental rats after anesthesia in order to exclude the influence of blood in the brain on the experimental results, and then euthanasia was performed by decapitation, and cortical samples <3 mm were collected at the edge of the damaged area (Figure 1B). The whole operation was conducted on ice. The collected brain tissues were immediately frozen and stored at -80 ^°^C until WB and ELISA. The brain tissues used to make paraffin sections were immersed in 4% paraformaldehyde. All tissue excision and selection methods were conducted by two pathologists blinded to the experimental conditions.

### WB analysis

Western blot analysis was performed as previously described (1). Brain tissues were homogenized and lysed in RIPA buffer supplemented with protease inhibitor mixture (Beyotime, China) and incubated on ice for 20 min. Then, the supernatant was collected by centrifugation at 12,000 *g* at 4 ^°^C for 20 min. The Pierce^TM^ BCA protein assay kit (Thermo Fisher, USA) was used to determine protein concentrations. Equivalent amounts (30 µg) of protein were loaded into each lane, isolated with 10% SDS-PAGE (Bio-Rad, USA), and then transferred onto a PVDF membrane (Millipore, USA). The membrane was sealed using QuickBlock^TM^ Western (Beyotime) and incubated at room temperature for 40 min, followed by overnight hybridization with the following primary antibodies in a 4 ^°^C refrigerator: HDAC3 (1:1,000, Cell Signaling Technology, USA, 3949S), Npas4 (1:1,000, Novus Biological, USA, NBP2-47252), LC3B (1:500, Boster Biological Technology, China, BM4827), Beclin-1 (1:500, Boster Biological Technology, China, BA3123-2), P62 (1:1,000, Abcam, UK, 5114S), IL-6 (1:1,000, ABclonal, USA, A0286), IL-1β (1:1,000, Abcam, UK, ab9787), TNF-α (1:1000, ABclonal, USA, A11534), albumin (1:1,000, Abcam, UK, ab207327), and GAPDH (1:5,000, Sigma, USA, G9545). GAPDH was used as the loading control. The next day, after washing with TBST thrice (10 min each time), the corresponding secondary antibodies were applied: anti-mouse IgG-HRP (1:1,000, Cell Signaling Technology, USA, 7076S) and goat anti-rabbit IgG-HRP (1:1,000, Invitrogen, USA, A16104) incubated at room temperature for one hour. Then, the slides were washed with TBST thrice for chemiluminescence (Millipore, USA, WBKLS0500) imaging. All WB strips were analyzed using ImageJ software (National Institutes of Health).

### Immunofluorescence staining

Immunofluorescence staining was performed as previously described (1). Paraffin sections of brain tissue were baked in an oven set at 70 ^°^C for 1 hr, then dewaxed in xylene, rehydrated in an alcohol gradient, and repaired with citric acid. These were permeabilized in 0.2% Triton-X solution and then washed with PBS (Beyotime, China) thrice (5 min each time). The brain sections were blocked with an immunoblocking solution for 30 min. Major antibodies: anti-mouse-HDAC3 (1:100, Cell Signaling Technology, USA, 3949S), anti-rabbit-NeuN (1:200, Abcam, UK, ab177487), anti-goat-Iba-1 (1:500, Abcam, UK, ab5076), and anti-rabbit-GFAP (1:500, Invitrogen, USA, PA1-9565) were added and incubated overnight in a 4 ^°^C refrigerator. After three washes in PBS, secondary fluorescent antibodies were added, and the slides were further incubated at room temperature for one hour. Finally, the sections were counterstained with a DAPI anti-fluorescent quenching solution (YEASEN, China), followed by imaging using a fluorescence microscope (OLYMPUS, Japan). The secondary antibodies used were as follows: donkey anti-mouse IgG antibody, Alexa Fluor 488 (1:1,000, Invitrogen, USA, A32766), donkey anti-goat IgG antibody, Alexa Fluor 555 (1:1,000, Invitrogen, USA, A21432), donkey anti-rabbit IgG antibody, and Alexa Fluor 555 (1:1,000, Invitrogen, USA, A32794).

### FJC staining

FJC staining was performed according to the manufacturer’s instructions (Biological Sensis, American) (2). Paraffin sections of brain tissues were baked in an oven set at 70 ^°^C Celsius for one hour, soaked in xylene, dehydrated using an ethanol gradient, and then washed twice for two minutes each with distilled water. The slices were then transferred to a solution containing one-tenth of solution C for 30 min. Then, the slides were washed with distilled water thrice and dried in a 60 ^°^C oven for ten minutes. This was followed by soaking in xylene for five minutes. After drying, the sections were mounted with a neutral resin (YEASEN, China), and images were captured using a fluorescence microscope.

### ELISA

ELISA kits were employed to detect inflammatory factors TNF-α (Boster Biological Technology, China, EK0526), IL-1β (Boster Biological Technology, China, EK0393), and IL-6 (Boster Biological Technology, China, EK0412) in brain tissues following the manufacturer’s instructions (Boster Biological Technology, China). The collected OD values were also converted into concentration values (7).

### Neurological scoring

All experimental rats were evaluated for neurological deficits 24 hr after SBI using the previously reported modified Garcia score (25), which included body proprioception, response to vibration and touch, symmetry of limb movement, spontaneous movement, lateral rotation, climbing ability, and walking using forelimbs. Each sub-test section was rated on a scale of 0-3, with a comprehensive score of 21 (no neurological impairment). The higher the score, the less the nerve damage. The whole quantitative scoring process was blind.

### Brain edema

Brain water content was assessed using the wet-dry method. The rats were decapitated after anesthesia, the brain tissues were separated and divided into two parts along the sagittal suture, and wet weight was immediately determined. The brains were then placed in a 100 ^°^C oven for 48 hr to determine dry weight (1). Brain water content was estimated using the following equation: 

Percentage of brain water content (%) = [(Wet weight - Dry weight)/Wet weight] × 100%.

### Statistical analysis

**Figure 2:**
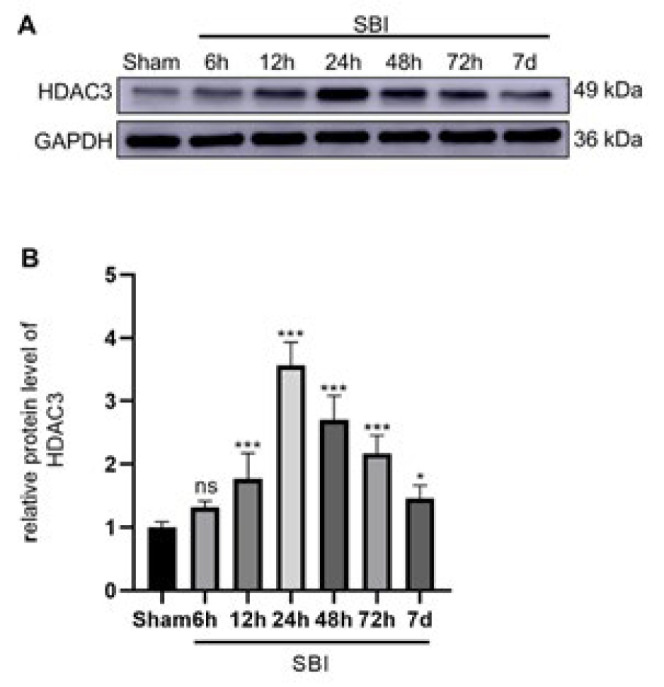
Protein expression of HDAC3 in the peripheral injury cortex after SBI in rats

All data were represented as mean±SD, and GraphPad Prism 8.0 (US) software was used for all statistical analysis. One-way ANOVA followed by Dunnett’s multiple comparisons test was utilized to compare the SBI and Sham groups for WB analysis, as shown in Figure 2 in Experiment 1. One-way ANOVA followed by Tukey’s multiple comparisons test was utilized to assess diﬀerences among groups in Figures 4 to 6 in Experiment 2. Immunofluorescence data were evaluated using unpaired t-test. *P*<0.05 was considered statistically significant. There were six rats in each group in Experiment 1. There were 12 rats in each group in Experiment 2; six rats in each group were assessed in terms of brain edema, and the remaining six rats in each group were used for other analyses. So neurological scoring was repeated 12 times, n=12 was described in Figure 6 legend; the remaining experiment items were repeated six times, n=6 was described in figure legends. 

## Results

### Protein expression levels of HDAC3 in the brain tissue after SBI

To determine the effect of brain surgery on HDAC3 expression in rats and its variation pattern, WB of the Sham and SBI groups at 6 hr, 12 hr, 24 hr, 48 hr, 72 hr, and 7 days after SBI was performed (Figure 2). We found that HDAC3 protein levels began to increase 12 hr after SBI and peaked after 24 hr. Subsequently, HDAC3 protein levels gradually decreased. However, at 7 days after SBI, HDAC3 expression remained higher than the Sham group.

### HDAC3 expression in cortical cells after SBI

**Figure 3:**
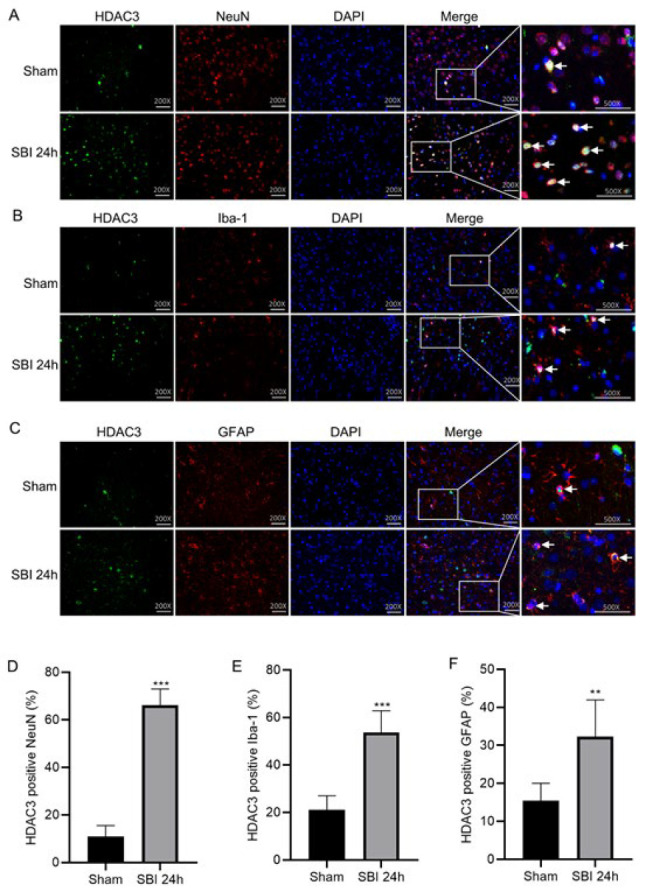
Double-immunofluorescent analysis of HDAC3 with neuron, microglia cell, and astrocytes glia cell in the peripheral injury cortex of rats

HDAC3 expression was evaluated by immunofluorescence staining using neuron marker (NeuN), microglia marker (Iba-1) or astrocyte marker (GFAP)(Figure 3). The number of HDAC3-positive neurons (Figure 3A), HDAC3-positive microglia (Figure 3B), and HDAC3-positive astrocytes (Figure 3C) increased in the 24-h post-SBI group compared with the Sham group, which coincided with the results of WB analysis.

### Effect of RGFP966 treatment on HDAC3 and Npas4 expression after SBI

**Figure 4:**
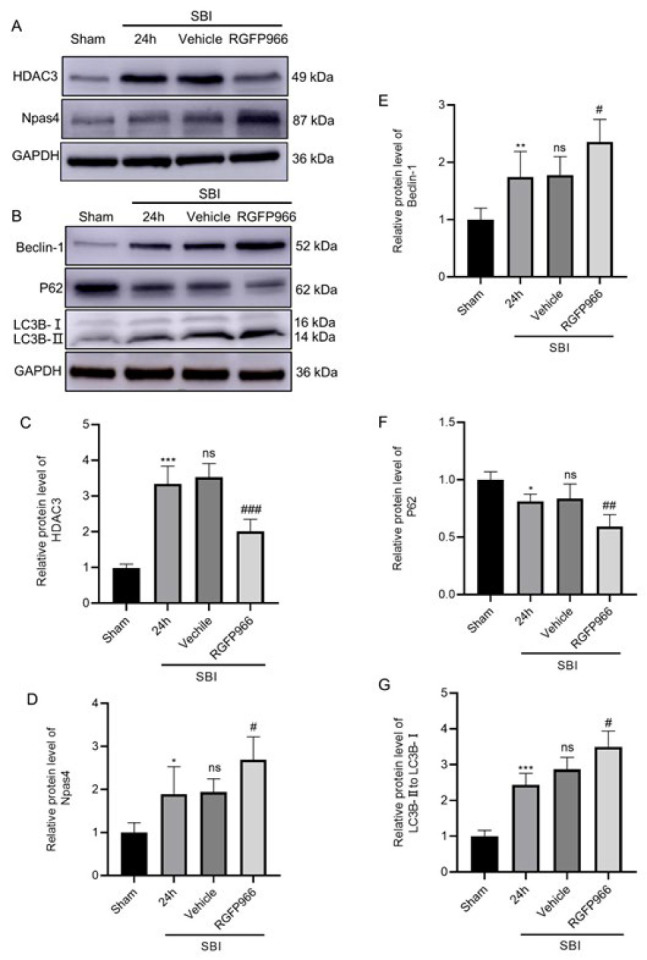
HDAC3, Npas4, and autophagy marker expression in brain tissues in each group with RGFP966 intervention in rats

WB showed that HDAC3 and Npas4 expression levels significantly increased in the SBI and SBI+Vehicle groups around the surgical area compared with the Sham group. No significant difference in HDAC3 and Npas4 expression was observed between the SBI and SBI+Vehicle groups. After RGFP966 treatment, HDAC3 expression in the SBI+RGFP966 group significantly decreased, while that of Npas4 further increased (Figure 4A, 4C, 4D).

### Effects of RGFP966 intervention on changes in autophagy markers and inflammation levels after SBI

**Figure 5:**
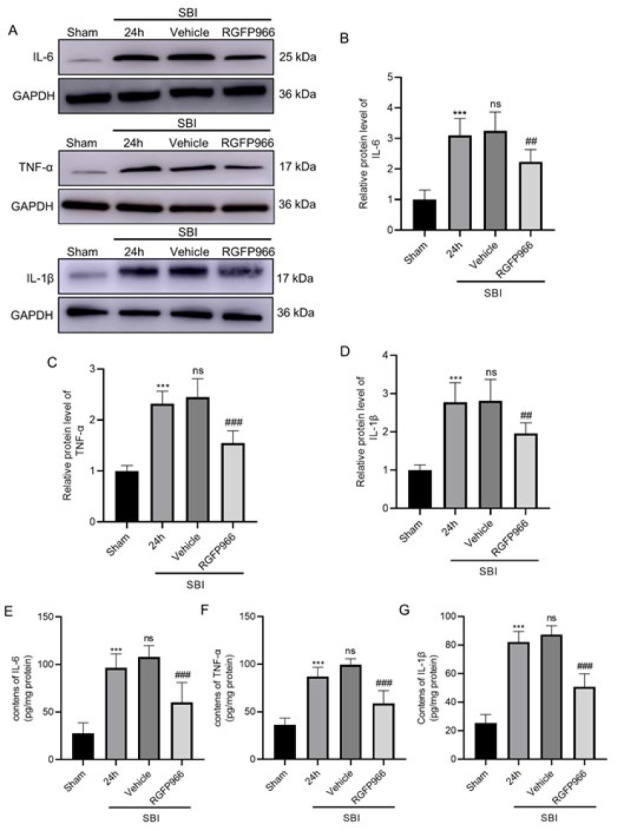
Expression level of inflammatory factors in each group after RGFP966 intervention in rats

WB results showed that compared with the Sham group, LC3B-II, and Beclin-1 expression levels significantly increased in the SBI and SBI+Vehicle groups around the surgical area, whereas P62 expression levels decreased. With RGFP966 treatment, LC3B-II and Beclin-1 expression in the SBI+RGFP966 group significantly increased compared to the SBI+Vehicle group, whereas P62 expression was significantly lower compared to the SBI+Vehicle group (Figure 4B, 4E-G). Compared with the Sham group, TNF-α, IL-1β, and IL-6 inflammatory cytokine expression levels significantly increased in the SBI and SBI+Vehicle groups. After RGFP966 treatment, inflammatory factor expression levels in the SBI+RGFP966 group were significantly lower than in the SBI+Vehicle group (Figure 5A-D). The results were further verified by Enzyme-Linked Immunosorbent Assay (ELISA)(Figure 5E-G).

### Effects of RGFP966 treatment on neurodegenerative death and brain edema in SBI

**Figure 6:**
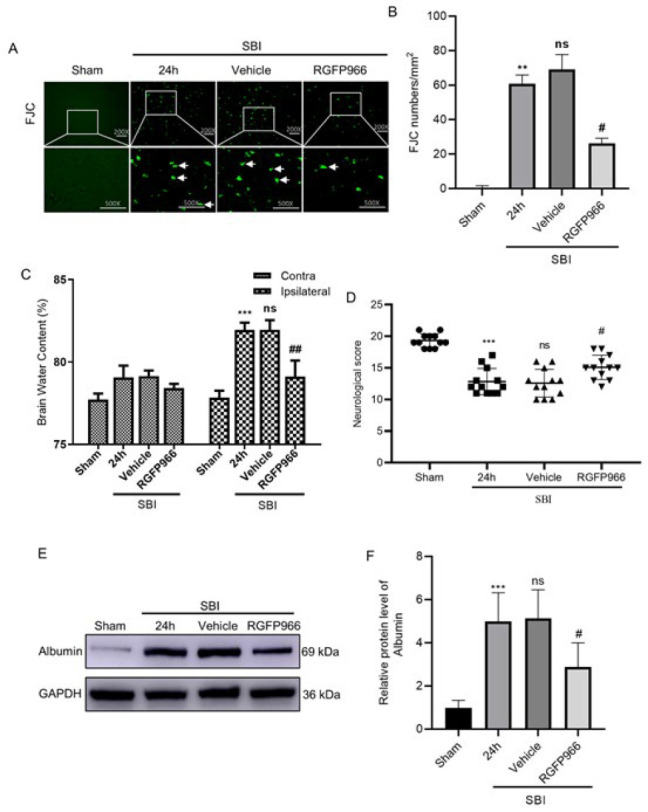
Nerve injury, neurological score, brain edema, and albumin expression level after RGFP966 intervention in rats

This study revealed that nerve cell degeneration in the SBI group surrounding the surgical area was more extensive than in the Sham group, whereas no significant difference was observed in the SBI and the SBI+Vehicle groups. Furthermore, after RGFP966 intervention, the degree of degeneration in the SBI+RGFP966 group was significantly lower than in the SBI+Vehicle group (Figure 6A, 6B). In addition, after SBI, brain edema of the damaged hemisphere significantly increased, which was significantly reduced with RGFP966 intervention, whereas that of the non-injured side did not significantly change between groups (Figure 6C). Albumin was used as an index to evaluate the integrity of the BBB. Compared with the Sham group, the albumin levels of the SBI and the SBI+Vehicle groups increased. After RGFP966 treatment, albumin levels significantly decreased (Figure 6E, 6F).

### Neurobehavioral score of SBI rats after RGFP966 intervention

The modified Garcia test neurobehavioral score was employed to evaluate the neurobehavior of rats in various study groups. Compared with the Sham group, the neurobehavioral scores of the SBI and SBI+Vehicle groups were significantly lower, whereas there was no difference between the SBI group and the SBI+RGFP966 group. Compared with the SBI+Vehicle group, the neurobehavioral score of the SBI+RGFP966 group was significantly improved (Figure 6D).

## Discussion

HDAC3 is a member of the HDAC family that participates in epigenetic modifications in the pathogenesis of various diseases. A previous study has shown that HDAC3 expression is up-regulated in neurons after traumatic brain injury (TBI) in SD rats (7), and our study has seen the same results. Our study proved that the expression of HDAC3 in the lesioned cortex after SBI was enhanced and expressed in neurons, microglia, and astrocytes. However, it was mainly reflected in neurons (Figure 3). In another study (28), HDAC3 expression was elevated in microglia after cerebral ischemia in mice but not in neurons or astrocytes. This differs from our study, possibly because we employed a different animal model. We explained for the first time the time evolution of HDAC3 in the rat SBI model: 12 hr after SBI, it began to rise, peaked at 24 hr after SBI (Figure 2), and then decreased. The above findings suggest that HDCA3 is involved in the pathologenesis of SBI. Previous studies have suggested that HDAC3 is up-regulated and mediates neurotoxicity in various animal models of acute central nervous system injury (26, 29). The specific inhibition of HDAC3 initiates a series of gene expression programs related to neuroprotection, thereby protecting the brain from damage (30, 31). Our research also confirmed this conclusion. At the peak of HDAC3 expression after SBI, we selected the highly selective HDAC3 inhibitor RGFP966 (27) to inhibit HDAC3. The results showed that HDAC3 expression decreased after treatment, autophagy increased, SBI inflammation significantly reduced, cerebral edema reduced, and nerve function improved.

Previous studies have confirmed that the neuroprotective transcription factor Npas4 is the transcriptional repression target of HDAC3 and promotes neuronal survival (20-22). Histone acetylation influences the transcription kinetics of the Npas4 gene, thereby affecting selective changes in neuronal gene expression and cell function (23). The expression level of Npas4 is related to the level of HDAC3 and histone acetylation (19). Some researchers have used ChIP to confirm that the Npas4 gene is the target of HDAC3-mediated transcriptional repression; they have found that HDAC3 occupancy was detectable at the promoter region of the Npas4 gene and co-expression of HDAC3 reduced the activity of the Npas4 promoters. They also overexpressed HDAC3 in cortical neurons using the adenovirus and found that Npas4 mRNA levels were down-regulated (20). The present study also obtained similar conclusions. Compared with the sham group, after SBI in rats, the self-regulating protective mechanism in the body was activated, and Npas4 expression significantly increased, which imparted neuroprotection. After inhibiting HDAC3, HDAC3 expression significantly decreased, but the expression of Npas4 was further enhanced (Figure 4A), and neurological function improved.

Fan *et al*. (23) found that overexpression of Npas4 can trigger the autophagy process of cultured cortical neurons, promote the formation of autophagosomes, and induce the up-regulation of LC3II, thereby protecting neurons. Similar to their conclusions, our study found that in rats with SBI, Npas4 expression increased in the brain tissues, autophagy markers LC3B-II and Beclin-1 were up-regulated, P62 was down-regulated, and autophagy levels increased. When HDAC3 inhibitors were administered, Npas4 expression significantly increased, autophagy was significantly up-regulated, and neurological function improved, indicating that autophagy after SBI has a neuroprotective function, and HDAC3 inhibition improves autophagy after SBI (Figure 4B). We hypothesize that this effect may be partly achieved by up-regulating Npas4.

Studies have shown that SBI is closely associated with inflammation (3, 6). The release of inflammatory factors can destroy nerve cells, leading to nerve cell necrosis and apoptosis (32-34). After brain injury, the expression of TNF-α, IL-6, IL-1β, and other inflammatory factors is up-regulated, which is closely related to the development of cerebral edema (16, 28). Npas4 has also been confirmed to be involved in the regulation of inflammation in the nervous system (24). Our study showed that compared with the sham group, inflammatory factors increased albumin secretion, and neuronal cell damage increased in the SBI group, indicating that SBI can cause neuroinflammation, brain edema, and neurological impairment (Figures 5 and 6). Inhibition of HDAC3 can reduce the expression of HDCA3 in lesions; the expression of Npas4 is significantly increased, the SBI-induced increase in inflammatory factors is reduced, and albumin levels also decrease. These results indicate that inhibiting the expression of HDCA3 can exert anti-inflammatory and neuroprotective effects. We hypothesize that Npas4 mediates this effect.

**Figure 7:**
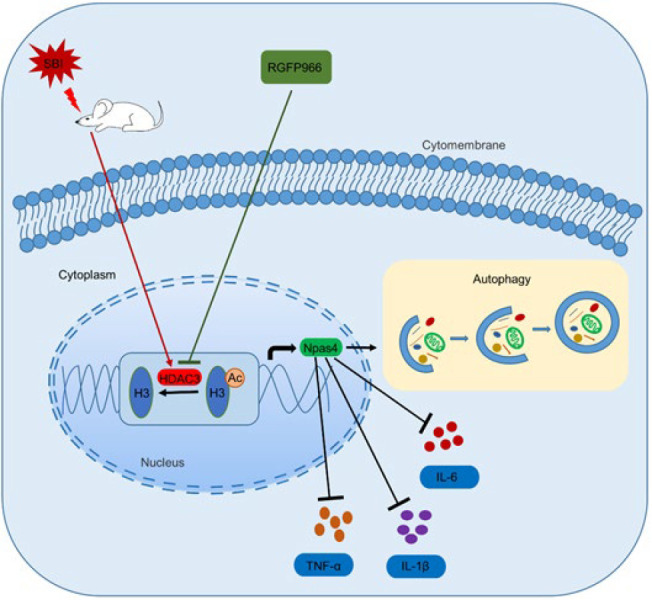
A possible mechanism by which RGFP966 influences SBI via the HDAC3/Npas4 pathway

Autophagy and inflammation are closely related (6). Autophagy and autophagy-related proteins comprise the basic components that control the inflammatory response. Furthermore, the expression of autophagy-related genes due to autophagy defects increases the inflammatory response elicited by human THP-1 macrophages (35). A previous study has suggested that in TBI rats, inhibition of autophagy up-regulates inflammatory factors and attenuates the neuroprotective effects associated with valproic acid treatment (7). The present study used another form to verify this conclusion. We enhanced the intensity of autophagy by inhibiting HDAC3 and increasing the expression of Npas4. At this time, the expression level of inflammatory factors decreased, and the outcome improved (Figure 7).

The present has several limitations. First, the sample size of this study is relatively small. Second, only male rats were included in this investigation; thus, we cannot investigate gender-related differences in HDAC3 expression post-SBI. Therefore, this point should be taken into consideration when interpreting our results. In addition, we did not study whether knocking out Npas4 will weaken or reverse the neuroprotective effect of HDAC3 inhibitors, thereby further confirming our above hypothesis on the role of Npas4. We are currently conducting further research to verify our results.

(A) Flow chart of Experiment 1. (B) Peripheral brain injury in SBI rats and the sham group was observed in the assay. (C) Flow chart of Experiment 2

(A) WB bands showing the protein levels of HDAC3 in the Sham group and SBI group at 6 hr, 12 hr, 24 hr, 48 hr, 72 hr, and 7 d. (B) Quantitative analysis of HDAC3. Protein levels were normalized to that of GAPDH. Each SBI group protein was normalized to the sham group. Data are expressed as the mean±SD; Statistical analyses were performed using One-way ANOVA followed by Dunnett’s post hoc test; ns *P>*0.05, *P<0.01, ***P<0.001 vs Sham group; n=6

HDAC3: Histone deacetylase 3; SBI: Surgical brain injury

Representative images of double-immunofluorescent staining using green-labeled HDAC3 and red-labeled NeuN (A)/Iba-1 (B)/GFAP (C) in the Sham and 24 hr post-SBI groups. Nuclei are indicated by DAPI (blue). The squares in merged images were magnified as representative pictures. The arrows show the colocalization of HDAC3 with neurons/microglia/astrocytes. Quantitative analysis of HDAC3 and NeuN (D)/Iba-1 (E)/GFAP (F) positive cells co-staining. The scale bar is 100 μm, the magnification is 200×. Data are expressed as the mean±SD; Unpaired t-test method was used for data analysis; **P<0.01, ***P<0.001 vs Sham group; n=6

HDAC3: Histone deacetylase 3; SBI: Surgical brain injury; DAPI: 4,6-diamino-2-phenyl indole

(A) WB was performed to assess HDAC3 and Npas4 expression levels in the Sham, SBI, SBI+Vehicle, and SBI+RGFP966 groups. (B) WB was performed to assess Beclin-1, P62, and LC3B-II expression levels in the Sham, SBI, SBI+Vehicle, and SBI+RGFP966 groups. Quantitative analysis of HDAC3 (C), Npas4 (D), Beclin-1 (E), and P62 (F). Protein expression was normalized to that of GAPDH. (G) Quantitative analysis of LC3B-II. Protein expression was normalized to that of LC3B-I. The data are expressed as the mean±SD; Statistical analyses were performed using One-way ANOVA followed by Tukey’s multiple comparisons test; *P<0.05, **P<0.01, ***P<0.001 vs Sham group; ns *P>*0.05 vs SBI group; ^#^*P<*0.05, ^##^*P<*0.01,^ ###^*P<*0.001 vs SBI+Vehicle group; n=6

SBI: Surgical brain injury; HDAC3: Histone deacetylase 3; Npas4: Neuronal Per-Arnt-Sim domain protein 4

(A) WB was used to detect IL-6, TNF-α, and IL-1β levels in the Sham, SBI, SBI+Vehicle, and SBI + RGFP966 groups. Quantitative analysis of IL-6 (B), TNF-α (C), and IL-1β (D). IL-6 (E), TNF-α (F), and IL-1β (G) expression in the brain tissues of different groups as detected with ELISA. The data are expressed as the mean±SD; Statistical analyses were performed using One-way ANOVA followed by Tukey’s multiple comparisons test; ***P<0.001 vs Sham group; ns *P>*0.05 vs SBI group; ^##^*P<*0.01, ^###^*P<*0.01 vs SBI+Vehicle group; n=6

SBI: Surgical brain injury; IL-6: Interleukin-6; TNF-α: Tumor necrosis factor-alpha‌; IL-1β: Interleukin-1 beta

(A, B) FJC staining in SBI rats after RGFP966 intervention. Arrowheads point to FJC-positive cells. The scale bar is 100 μm, the magnification is 200×. n=6. (C) The brain water content in the bilateral hemispheres of various groups was determined using the wet-dry method. n=6. (D) Neurological behavioral scores of SBI rats after RGFP966 treatment. n=12. (E) WB was performed to assess albumin levels in the Sham, SBI, SBI+Vehicle, and SBI+RGFP966 groups. n=6. (F) Quantitative analysis of albumin. n=6. The data are expressed as the mean±SD; Statistical analyses were performed using One-way ANOVA followed by Tukey’s multiple comparisons test; **P<0.01, ***P<0.001 vs Sham group; ns *P>*0.05 vs SBI group; #*P<*0.05, ^###^*P<*0.001 vs SBI+Vehicle group

SBI: Surgical brain injury; FJC: Fluoro-Jade C

After SBI, HDAC3 expression increased and inflammation levels were significantly enhanced. The enhanced autophagy protection function was not enough to offset the inflammatory damage, resulting in brain edema and nerve injury. After specific inhibition of HDAC3 by RGFP966, Npas4 expression significantly increased, autophagy was enhanced, inflammation levels decreased, and brain edema and nerve injury were reduced. H3: histone 3; AcH3: Acetylated histone 3

## Conclusion

This study verified that HDAC3 plays a role in the complex pathogenesis of SBI in rats. Inhibiting HDAC3 can improve neuroinflammation and autophagy after SBI by imparting a neuroprotective effect mediated by the up-regulation of Npas4. This study suggested that HDAC3 inhibition may be a potential target for SBI.

## Data Availability

The data are available from the corresponding author.

## Declaration

The authors confirm that no paper mill was used and that all data were generated in-house.
